# Thermal Performance of Heat Sink Filled with Double-Porosity Porous Aluminum Skeleton/Paraffin Phase Change Material

**DOI:** 10.3390/mi15060806

**Published:** 2024-06-20

**Authors:** Shufeng Huang, Chuanshun Long, Zhihan Hu, Yingshuai Xu, Bin Zhang, Changjian Zhi

**Affiliations:** 1 School of Mechanical and Electronic Engineering, East China University of Technology, Nanchang 330013, China; 2 Jiangxi Engineering Province Engineering Research Center of New Energy Technology and Equipment, East China University of Technology, Nanchang 330013, China

**Keywords:** double-porosity porous aluminum skeleton, phase change material, heat sink, thermal management

## Abstract

Phase change materials (PCMs) are used to cool high-power-density electronic devices because of their high latent heat and chemical stability. However, their low thermal conductivity limits the application of PCMs. To solve this problem, a double-porosity porous aluminum skeleton/paraffin phase change materials (DPAS/PCM) was prepared via additive manufacturing and the water-bath method. The thermal performance of the DPAS/PCM heat sink (HS) was experimentally investigated to examine the effects of the positive- and reverse-gradient porosity structures of the DPAS/PCM. The results show that a positive-gradient porosity arrangement is more conducive to achieving a low-temperature cooling target for LED operation. In particular, the temperature control time for the positive gradient porosity structure increased by 4.6–13.7% compared with the reverse gradient porosity structure. Additionally, the thermal performances of uniform porous aluminum skeleton/paraffin (UAS) and DPAS/PCMs were investigated. The temperature control effect of the DPAS/PCM was better than that of the UAS/PCM HS at high critical temperatures. Compared with the UAS/PCM HS, the temperature control time of the DPAS/PCM HS is increased by 7.8–12.5%. The results of this work show that the prepared DPAS/PCM is a high-potential hybrid system for thermal management of high-power electronic devices.

## 1. Introduction

With the development trend of high performance, miniaturization and functional integration of modern optoelectronic devices such as light emitting diodes (LED) [1,2,3] and lasers [4], a large amount of heat is generated inside electronic components, which increase the operating temperature that affects the life and reliability of the modern electronic devices [5]. Different efficient heat dissipation techniques for electronic devices have been proposed, including substrates with high thermal conductivity [6,7], pool boiling [8], jet impingement cooling [9], and heat pipes [10]. Phase change material (PCM) has significant potential to cool high heat-generating electronic components due to being readily available, inexpensive, compatible with other materials, chemically inert, recyclable, and thermally stable [11]. Therefore, phase change materials are widely used in LED [12], energy-saving buildings [13], intelligent equipment [14,15], aerospace [16] and other fields. However, due to the inherently low thermal conductivity of PCM, it is a challenge to achieve efficient heat dissipation in temperature management, which limits their further engineering applications [17,18]. To address this drawback, various methods have been developed to improve the thermal conductivity of PCM, including inserting metal fins [19,20], adding high thermal conductivity nanoparticles [21], embedding metal foams [22,23] and microencapsulating [24].

Among the many methods for enhancing thermal conductivity, the introduction of inner fins has attracted attention in recent years. Fok et al. [25] conducted thermal management on portable electronic devices using heat sinks (HSs) comprising fins of n-eicosane PCM; the results showed that the HSs with PCM fins were more effective than those without fins. Other studies have also been conducted that employed inner fins to improve the thermal conductivity of PCMs [26,27]. Although inner fins can improve the thermal performance of a PCM-based thermal control system, they lead to an increase in the size, weight, and manufacturing cost of the system.

Accordingly, another reinforcement technique involving the addition of nanoparticles has been attempted to solve the problem of low thermal conductivity. Owing to their large specific surface area, nanoparticles have been used to increase heat dissipation in asphalt [28] as well as enhance the thermal properties of cement mortar [29] and nanofluids [30]. Although the introduction of nanoparticles into a PCM reduces the weight of the thermal control system, the nanoparticles can agglomerate, which reduces the cooling effect of the PCM. Further, nanoparticles increase the viscosity of PCMs, which limits convective heat transfer.

In addition to the abovementioned methods, embedding PCM into porous materials with high thermal conductivity has proven to be the most effective way to solve the low thermal conductivity of PCMs [31]. Baby and Balaji [32] proposed a copper foam (CuF)-PCM HS to dissipate heat from electronic components. The results showed that the maximum enhancement ratio is 3 when the set point temperature is 52 °C; the effect of orientation on increasing heat transfer performance is not significant. Rehman and Ali [33] investigated the addition of copper foam and iron-nickel foam to PCM to cool electronic devices and chose RT-35HC as the PCM. As a result, the temperature of the HS with copper foam PCM was 5–6 °C lower than that with the Fe-Ni foam PCM. The same authors [34] studied the effect of the PCM volume on thermal performance. Compared with pure nickel foam, the temperature of CoF-HS with 0.8 PCM volume fraction was reduced by 26%. Wang et al. [12] investigated an HS without a PCM, an HS with a pure PCM, and an HS with a porous metal sintered felt (PMFSF)-PCM. The addition of the PMFSF enhanced the heat transfer of the PCM and reduced the heat source temperature. It can be clearly seen from the literature that metal foams (such as copper, iron, aluminum, and nickel) and non-metallic foams (such as carbon and graphene) can be used with PCM to improve the efficiency of PCM-based thermal control modules [31,32,33,34]. However, the limited improvements in thermal conductivity are insufficient to satisfy the increasing thermal storage and dissipation requirements of high-power electronics.

Considering the above situation, it is necessary to develop a new PCM with a high latent that is suitable for cooling high-power electronics. Considering the operating temperatures of high-power electronic devices, very little work has been conducted using double-porosity porous skeleton/paraffin PCM. This study proposes a novel double-porosity porous aluminum skeleton/paraffin PCM (DPAS/PCM) based on additive manufacturing and a water bath method. The preparation and experimental system are described in detail in Section 2. Furthermore, the transient thermal performance of the DPAS/PCMs is experimentally studied in Section 3. Finally, the thermal properties of the double-porosity and uniform HSs were compared.

## 2. Materials and Methods

### 2.1. Experimental Platform

**Figure 1:**
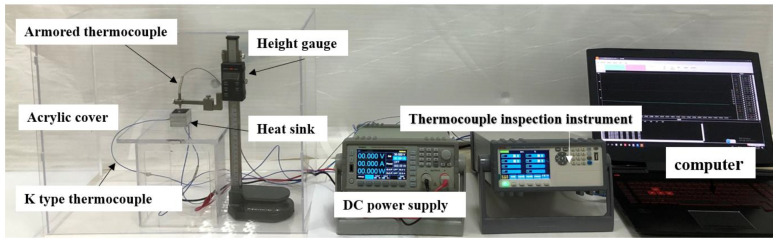
Physical image of experimental platform.

As shown in Figure 1, the experimental platform comprised an HS with LEDs (Cree lnc., Durham, NC, USA), an altimeter (Mitutoyo, Shanghai, China), a DC power supply (Guangdong Henghuiyuan Electronics Co., Ltd., Guangdong, China), a temperature acquisition system (Changzhou Jinailian Electronic Technology Co., Ltd., Changzhou, China), a computer (China Hewlett-Packard Co., Ltd., Beijing, China), and a Plexiglas box. The HS was placed in the plexiglass box to prevent heat exchange with its surroundings. The temperature detection unit consisted of K-type thermocouples, a temperature detector, and a computer. Two K-type thermocouples were glued to the LED base plate midpoint (T1) and cavity side midpoint (T2) to analyze the instantaneous temperatures T1 and T2. Another K-type thermocouple was secured and positioned using an altimeter to test the T3 instantaneous temperature. The LEDs were powered by a DC power supply, and four power levels of 11.7, 14.7, 17.7, and 21.3 W were applied to each HS to test the transient performance of the HSs. The specific experimental steps are as follows: (1) Pre-regulate the DC power supply to output the desired power. (2) Start the temperature acquisition system and turn on the power to apply constant power to the LEDs to heat the DPAS/PCM heat sink. (3) Turn off the power to end the heating process when T1 reaches the limit temperature of 90 or 100 °C. (4) Heat sink naturally cools until the system temperature drops below 27 °C and stops the temperature acquisition system. In addition, the temperature control experiment was repeated three times for each sample at each heating power, and the average value was obtained as the experimental result. Specifically, the employed K-type thermocouples had an accuracy of 0.1 °C, and the input power accuracy was ±0.1 W.

### 2.2. LED Heat Sink (HS) Cooling Device

**Figure 2:**
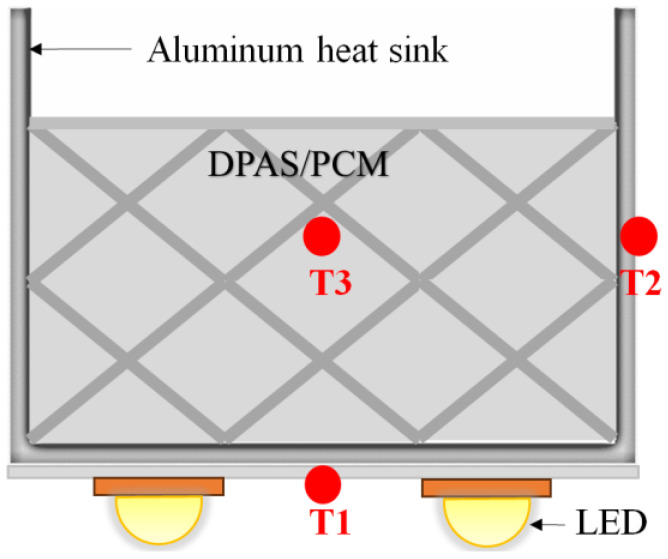
Diagram of a heat sink (HS) constituting a double-porosity porous aluminum skeleton/paraffin phase change material.

A light-emitting diode (LED) HS-cooling device (LED-HD) stores the heat generated by the LED during operation as heat and emits the remaining heat through natural convection on the outer surface of the aluminum cavity and radiation heat dissipation of the HS, ensuring that the LED operates below the limit temperature. As shown in Figure 2, the device consists of a cubic aluminum cavity, a DPAS/PCM, and an LED light heat source. The heat storage of the LED-HD is mainly provided by the inner DPAS/PCM. The dimensions of the aluminum cavity were as follows: 30 mm in length, 30 mm in width, 25 mm in height, and 2 mm in wall thickness. The LED heat source was fixed in the middle of the bottom surface using a thermally conductive two-sided adhesive, and the power of the LED was 8–35 W. As shown in Figure 2, the DPAS/PCM HS has three temperature measuring points: the bottom (T1), side (T2), and HS center (T3). To test the central temperature of the AS/PCM, a hole with a depth of 10 mm and a diameter of 1 mm was drilled at the center of the DPAS/PCM. In addition, the DPAS/PCM HS was equipped with three temperature-measuring points, as shown in Figure 2: the center of the HS (T3), bottom (T1), and side (T2). To obtain the central temperature of the DPAS/PCM, a small hole with a depth of 10 mm and a diameter of 1 mm was drilled in the center of the DPAS/PCM with a drill bit. The safe operating temperature range is 45 °C to 100 °C.

### 2.3. Preparation of Double-Porosity Porous Aluminum Skeleton/Paraffin Phase Change Material

**Figure 3:**
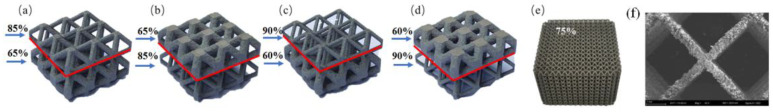
Porous aluminum skeleton samples: (**a**) DPAS6585, (**b**) DPAS8565, (**c**) DPAS6090, (**d**) DPAS9060, (**e**) uniform porous aluminum skeleton, (**f**) the morphology of DPAS6585/PCM.

The preparation of the DPAS/PCM was divided into two steps: fabrication of a double-porosity porous aluminum skeleton (DPAS) using the additive manufacturing method and preparation of the DPAS/PCM using the water bath method. Figure 3 shows the four types of DPAS samples based on the AM. Figure 3a shows a DPAS with 65% porosity at the bottom and 85% porosity at the top, named DPAS6585. The arrangement of DPAS6585, with low porosity in the first layer and high porosity in the second layer, was defined as a positive-gradient porosity. Conversely, DPAS8565 is called reverse gradient porosity. Figure 3f shows that the DPAS has a three-dimensional connected pore structure and a rough surface, which is conducive to natural convection heat transfer. For comparative analysis, a uniform porous aluminum skeleton (UAS) with the same overall porosity of 75% was also prepared, as shown in Figure 3e. For DPAS6585 and DPAS8565, the filament size of the 65% porosity layer is 3.01 mm, and the filament size of the 85% porosity layer is 1.86 mm. For DPAS6090 and DPAS9060, the filament size of the 60% porosity layer is 3.25 mm, and the filament size of the 85% porosity layer is 1.50 mm. As shown in Figure 3f, the printed skeleton has a rough surface topography.

**Figure 4:**
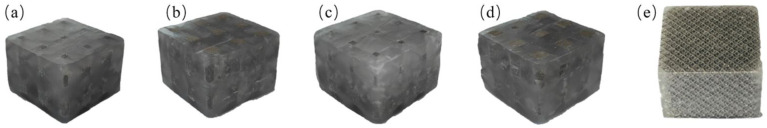
DPAS/PCM samples: (**a**) DPAS6585/PCM, (**b**) DPAS8565/PCM, (**c**) DPAS6090/PCM, (**d**) DPAS9060/PCM, (**e**) UAS/PCM.

The DPAS/PCMs were prepared in four steps based on the water bath method. (1) A heat-resistant soft silicone mold was placed in a beaker, an appropriate amount of paraffin was weighed and placed into the silicone mold, and then DPAS was placed on the paraffin. (2) The above beaker was placed in a water bath; the temperature of the water bath was adjusted to about 80 °C and maintained for a period of time, and the DPAS was completely immersed in the paraffin. (3) After being kept at 80 °C for a period of time, the power supply was turned off, and the sample cooled naturally until the paraffin in the beaker solidified. (4) Excess paraffin was removed from the surfaces of the samples to obtain samples with dimensions of 30 × 30 × 20 mm, as shown in Figure 4. In addition, Figure 4e shows that a uniform porous aluminum skeleton/paraffin PCM (UAS/PCM) was prepared for comparison. As shown in Table 1, the thermal conductivity of the AS/PCM has been tested based on the transient plane heat source method.

## 3. Results and Discussion

### 3.1. Thermal Performance Analysis of a Typical DPAS/PCM HS

**Figure 5:**
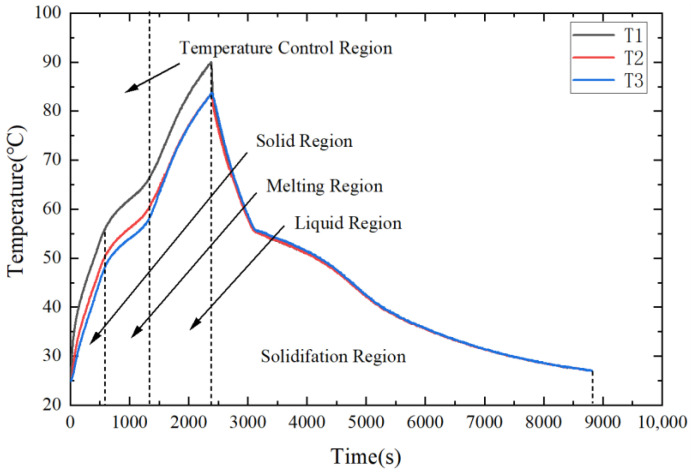
Transient temperature of DPAS/PCM HS at an input power of 11.7 W.

Figure 5 displays the transient temperature of the DPAS6585/PCM HS at the input power of 11.7 W. From Figure 5, the transient temperature curve can be divided into four segments: solid, melting, liquid, and solidification. The average velocity *V* of T1 is defined as *V* = Δ*T*/Δ*t*, where Δ*t* is the duration of the corresponding interval, and Δ*T* is the change in temperature T1 during Δ*t*.

The average temperature increase rates of T1 in the solid and liquid regions were 0.0515 and 0.0225 K/s, respectively. Meanwhile, the average temperature rise rate in the melting region was only 0.0136 K/s, which is 0.604 times that in the liquid-state region and 0.264 times that in the solid-state region. Thus, the latent heat of the phase transition inhibits the temperature increase of the LED in the melting region.

### 3.2. DPAS/PCM during the Heating Stage

**Figure 6:**
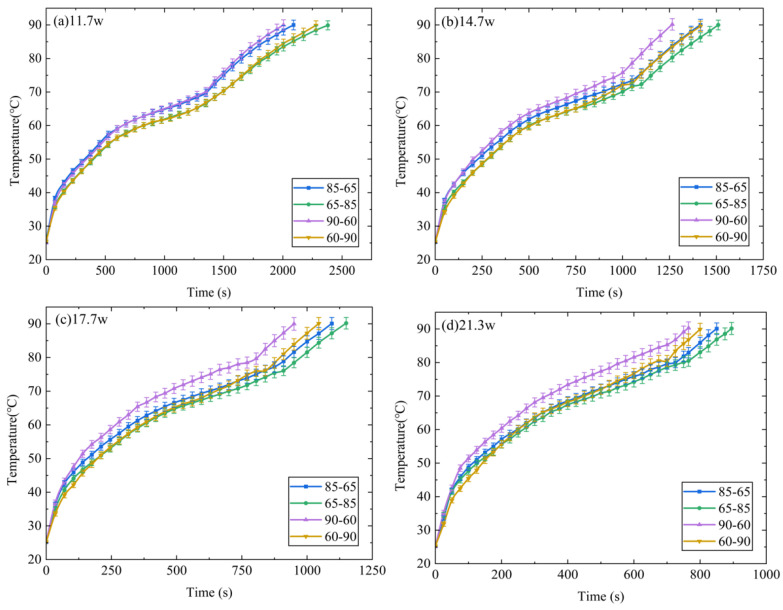
Comparison between T1 of different DPAS/PCM HSs during the heating stage: (**a**) 11.7 W, (**b**) 14.7 W, (**c**) 17.7 W, (**d**) 21.3 W. The T1 of the positive gradient porosity heat sink is smaller than that of the reverse gradient porosity heat sink (T1_60–90%_ < T1_90–60%_, T1_65–85%_ < T1_85–65%_).

Figure 6 presents a comparison between temperatures T1 of the DPAS/PCM HSs (DPAS6585/PCM, DPAS8565/PCM, DPAS6090/PCM, DPAS9060/PCM) during the heating phase. In the solid-state phase, T1 of the DPAS/PCM HS with a positive gradient porosity was smaller than that of the DPAS/PCM HS with a reverse gradient porosity; specifically, T1_60–90%_ < T1_90–60%_, T1_65–85%_ < T1_85–65%_. Furthermore, T1_60–90%_ < T1_90–60%_, T1_65–85%_ < T1_85–65%_ is also apparent in the melting stage. This can be attributed to the following reasons: the thermal conductivity of DPAS6585/PCM is 11.99% higher than that of DPAS8565/PCM based on the thermal conductivity results. In addition, the thermal conductivity of DPAS6090/PCM is 34.94% higher than that of DPAS9060/PCM. Therefore, T1 for PCMs with positive-gradient porosity is smaller than that for PCMs with negative-gradient porosity. In the liquid phase, the temperature rise rate of the four DPAS/PCMs is basically the same and ultimately reaches 90 °C.

**Figure 7:**
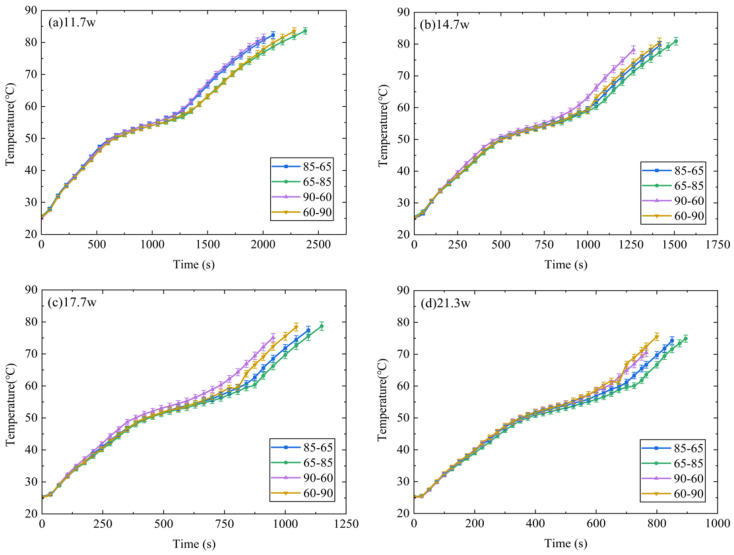
Comparison between T3 of different DPAS/PCM HSs during the heating stage: (**a**) 11.7 W, (**b**) 14.7 W, (**c**) 17.7 W, (**d**) 21.3 W. T3 with a porosity difference of 20% is smaller than that with a porosity difference of 30% at high power.

Figure 7 presents a comparison between temperatures T3 of the different DPAS/PCM HSs during the heating stage. In the solid phase, no significant difference is observed in the transient temperature for all the DPAS/PCMs because the overall porosity of the DPAS/PCMs was the same at 75%, while the heat transfer was dominated by the aluminum skeleton in the solid phase. After entering the melting stage, a significant gap occurs between T3 with the porosity difference of 20% and T3 with the porosity difference of 30%, T3_60–90%_ > T3_65–85%_, T3_90–60%_ > T3_85–65%_, which increased as the power increased. As the DPAS/PCM enters the liquid phase, T3_70–80%_ gradually approaches T3_80–70%_, and the difference in T3 decreases because natural convection dominates the heat transfer. Because high-porosity DPAS/PCMs have larger pores, which are conducive to natural convection.

**Figure 8:**
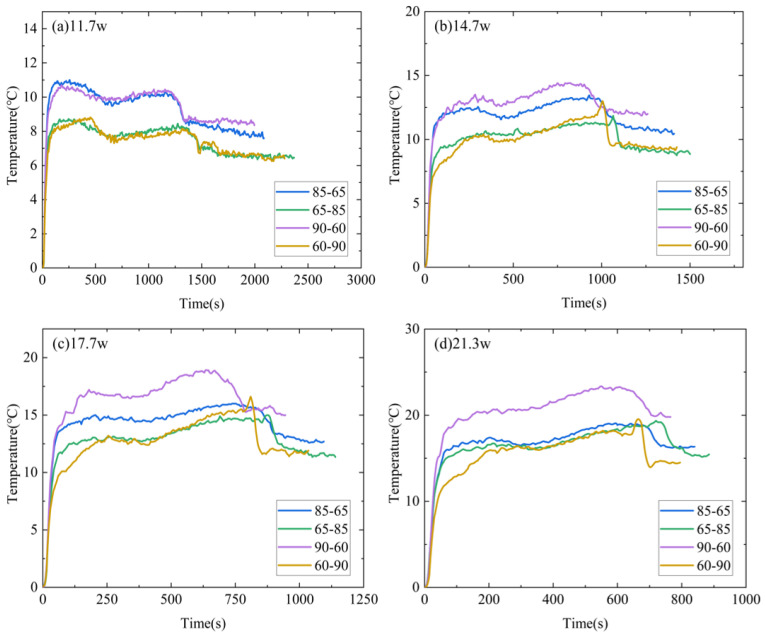
Comparison between ΔT2 of different DPAS/PCM HSs at the heating stage: (**a**) 11.7 W, (**b**) 14.7 W, (**c**) 17.7 W, (**d**) 21.3 W. The ΔT2 of the positive gradient porosity heat sink is smaller than that of the reverse gradient porosity heat sink (ΔT2_60–90%_ < ΔT2_90–60%_, ΔT2_65–85%_ < ΔT2_85–65%_).

Figure 8 compares the temperature difference ΔT2 for the various DPAS/PCMs. The ΔT2 value for positive gradient porosity is lower than ΔT2 for reverse gradient porosity at any power. For example, at the power of 11.7 W, ΔT2_65–85%_ is approximately 2 °C lower than ΔT2_85–65%_; ΔT2_60–90%_ and ΔT2_90–60%_ are also 2° lower. The results show that a positive-gradient porosity arrangement is more conducive to achieving a low-temperature cooling target for LED operation.

### 3.3. Temperature Control Time Analysis of DPAS/PCM

**Figure 9:**
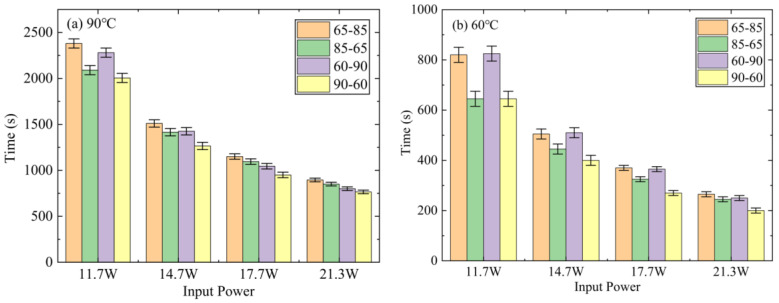
Temperature control time of DPAS/PCM HS at critical temperatures of (**a**) 90 °C and (**b**) 60 °C. At critical temperatures of 90°C, DPAS65-85/PCM has the longest temperature control time.

Figure 9a displays a comparison of the temperature control time for the different DPAS/PCM HSs, with 90 °C as the critical temperature. As shown in Figure 9, all DPAS/PCM HSs exhibit a decrease in temperature control time with increasing power, and the temperature control time for HSs with the porosity difference of 20% (DPAS6585/PCM, DPAS8565/PCM) is significantly longer than that for HSs with the porosity difference of 30% (DPAS6090/PCM, DPAS9060/PCM). In addition, the HS temperature control time is longer for positive-gradient porosity. From Figure 9a, the temperature control time of DPAS6090 increased by 4.6–13.7% compared with DPAS9060. At the critical temperature of 90 °C, DPAS6585/PCM exhibits the best temperature control effect. Taking the power of 11.7 W as an example, the temperature control times of DPAS6585/PCM were 13.8%, 4.4%, and 18.7% higher than those of DPAS8565/PCM, DPAS6090/PCM, and DPAS9060/PCM, respectively. Meanwhile, at the critical temperature of 60 °C, the DPAS6090/PCM presented the best temperature control at the power of 11.7 W. The temperature control time of the DPAS6090/PCM increased by 0.06%, 27.9% and 27.9% compared to the DPAS6585/PCM, DPAS9565/PCM, and DPAS9060/PCM, respectively. Therefore, to realize the low-temperature operation of LED electronics, a DPAS/PCM HS with a positive gradient porosity and a smaller gradient porosity difference is preferred for temperature control.

## 4. Comparative Study of DPAS/PCM and UAS/PCM

### 4.1. Comparison at Heating Stage

**Figure 10:**
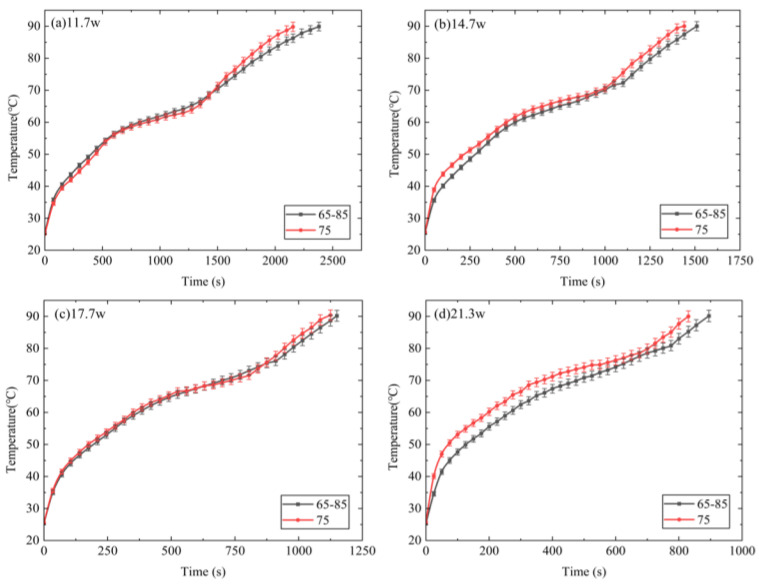
Comparison of T1 for DPAS/PCM and UAS/PCM HSs during the heating stage considering powers of (**a**) 11.7 W, (**b**) 14.7 W, (**c**) 17.7 W, (**d**) 21.3 W. The temperature control time for the DPAS/PCM heat sink was longer than for the UAS/PCM heat sink.

Figure 10 presents a comparison of T1 for the DPAS/PCM and UAS/PCM HSs during the heating stage. In the solid-state phase, T1 of the DPAS/PCM HS is lower than that of the UAS/PCM HS at a higher power. In addition, T1 of the DPAS/PCM HS is lower than that of the UAS/PCM HS at a higher power during the melting stage. The reason for the lower T1 of the DPAS6585/PCM at high power is because, compared with the UAS/PCM HS with 75% porosity, the positive gradient porosity structure of the DPAS6585/PCM provides high thermal conductivity in the lower part and relatively good natural convection in the upper part. Consequently, the combination of these two factors results in the lower T1 for the DPAS/PCM HSs. In addition, based on the thermal conductivity results, the thermal conductivity of DPAS6585/PCM is 2.6% higher than that of UAS/PCM. Therefore, T1 of the DPAS/PCM HS is lower than that of the UAS/PCM HS. The temperature control time for the DPAS/PCM HS is longer than that for the UAS/PCM HS because a larger volume of paraffin with higher porosity was used in the upper layer of the DPAS/PCM HS.

**Figure 11:**
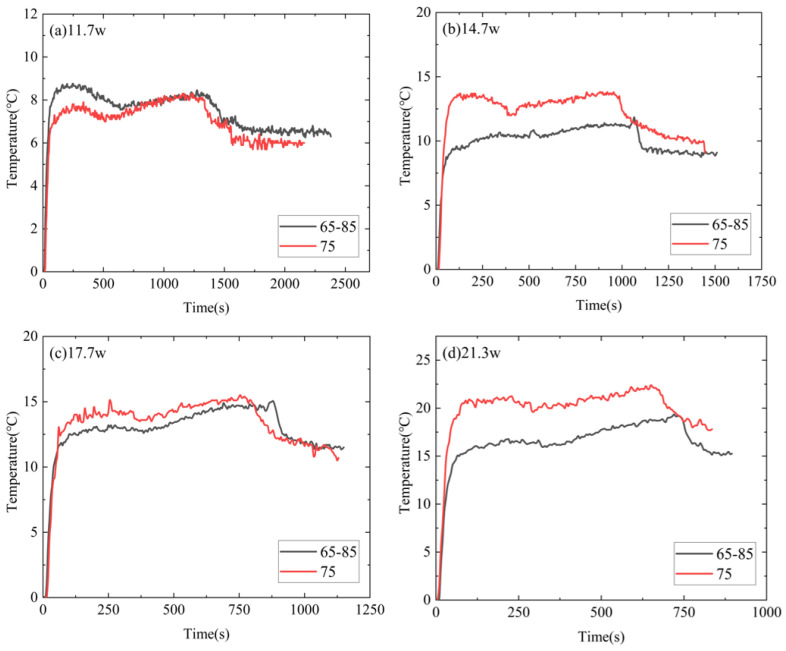
Comparison of ΔT2 for DPAS/PCM and UAS/PCM HSs during the heating stage at powers of (**a**) 11.7 W, (**b**) 14.7 W, (**c**) 17.7 W, (**d**) 21.3 W. The DPAS/PCM heat sink is more suitable for LEDs operating at high power.

Figure 11 compares the temperature difference ΔT2 for the DPAS/PCM and UAS/PCM HSs. It can be seen that ΔT2_65–85%_ < ΔT2_75%_ at high power; ΔT2_65–85%_ is 1~5 °C lower than ΔT2_75%_, which indicates that DPAS/PCM HS is more suitable for LEDs operating at high power.

### 4.2. Comparison at Cooling Stage

**Figure 12:**
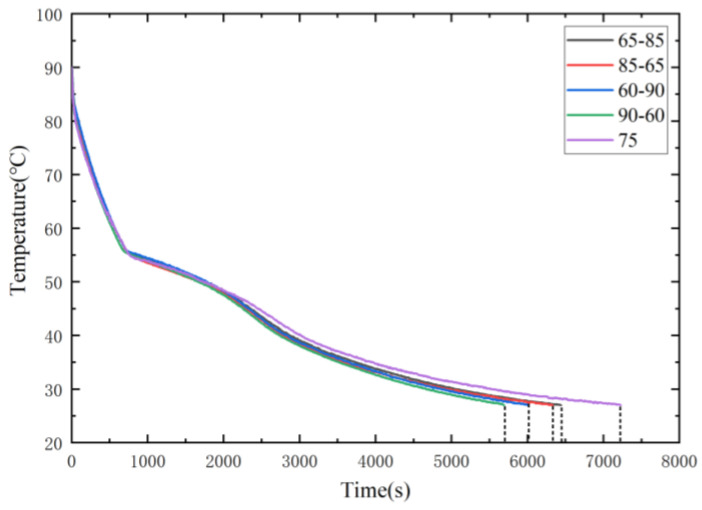
Comparison of cooling curves (T1) for DPAS/PCM and UAS/PCM HS. The cooling process of the DPAS/PCM heat sink is shorter than that of the UAS/PCM heat sink.

Figure 12 presents a comparison of the cooling curves (T1) for the DPAS/PCM and UAS/PCM HSs. The cooling time of the UAS/PCM HS with 75% porosity was 7225 s. The cooling times for the DPAS6585/PCM, DPAS8565/PCM, DPAS6090/PCM, and DPAS9060/PCM HSs were 6440, 6330, 6005, and 5690 s, respectively. Moreover, the results show that the cooling process of the DPAS/PCM HS was shorter than that of the UAS/PCM HS. The cooling time of the DPAS/PCM HS is 12.19~26.98% less than that of the UAS/PCM HS. In addition, the cooling time of the HS with reverse-gradient porosity was shorter than that of the HS with positive-gradient porosity. For example, the cooling time of the DPAS8565/PCM HS is 100 s shorter than that of the DPAS6585/PCM HS, while that of the DPAS9060/PCM HS is 315 s shorter than that of the DPAS6090/PCM HS.

### 4.3. Comparison of Temperature Control Times

**Figure 13:**
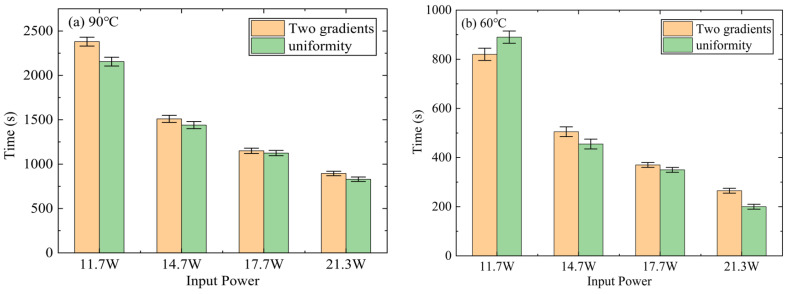
Temperature control time comparison between DPAS/PCM and UAS/PCM at critical temperatures of (**a**) 90 °C and (**b**) 60 °C. The temperature control time of the double-porosity heat sink is better than that of the uniformity heat sink under high-power conditions.

Figure 13 presents a comparison of the temperature-control times for the DPAS/PCM and UAS/PCM HSs. As shown in Figure 13, the temperature control time of the DPAS/PCM HS was significantly prolonged compared with that of the UAS/PCM HS. At the critical temperature of 90 °C, the temperature control effect of the DPAS/PCM is better than that of the UAS/PCM HS, but the gap gradually reduces with increasing power. The temperature control time of the DPAS/PCM increased by 12.5% at 11.7 W compared with that of the UAS/PCM HS, whereas it only increased by 7.8% at 21.3 W. Meanwhile, at the critical temperature of 60 °C, the gap between DPAS/PCM and UAS/PCM HSs became larger with increasing power. Uniform HSs are preferred under low-power conditions; i.e., at 11.7 W, the temperature control time of DPAS/PCM HSs is 7.8% shorter than that of UAS/PCM HSs. Under high-power conditions, the DPAS/PCM HS is preferred, and the temperature time of the DPAS/PCM HS is 32.5% longer than that of UAS/PCM HS at 21.3 W, for example. Therefore, UAS/PCM or DPAS/PCM HSs are selected in engineering applications depending on the power and operating temperatures of the electronic devices.

## 5. Conclusions

(1) A DPAS/PCM is proposed to solve the problem of low thermal conductivity of the PCM, and the positive-gradient porosity structure of the DPAS/PCM is more conducive to achieving low-temperature LED operation, mainly because of the high heat conduction near the heat source and the lowest resistance of natural convection in the upper part;

(2) The positive- and reverse-gradient porosity structures of the DPAS/PCM are very sensitive to the thermal performance. The LED temperature of the DPAS/PCM HSs with a positive-gradient porosity was lower than that of the DPAS/PCM HSs with a reverse-gradient porosity;

(3) Compared with the UAS/PCM HS, the DPAS/PCM HS is preferred under high-power conditions, and the temperature of the DPAS/PCM HS was 32.5% higher than that of the UAS/PCM HS at 21.3 W.

The results will provide some reference for the optimal design of the porous skeleton, which is the research to be focused on for future work.

## Figures and Tables

**Table 1:** Thermal conductivities of AS/PCMs.

AS/PCM	DPAS6585/PCM	DPAS8565/PCM	DPAS6090/PCM	DPAS9060/PCM	UAS/PCM
*k* (W/m·K)	12.23	10.92	11.74	8.7	11.92

## Data Availability

All data supporting the results of this study have been included in this article.
